# Epidemiological Characteristics and Occupational Impact of Organic Solvent-Induced Psychosyndromes

**DOI:** 10.1192/j.eurpsy.2025.1805

**Published:** 2025-08-26

**Authors:** G. Ben Rejeb, N. Kammoun, S. Ernez, S. Haj Ali, S. Fehri

**Affiliations:** 1 Tunisian Occcupational Safety and Health Institute; 2 University Tunis El Manar, Faculty of medicine of Tunis, Tunis, Tunisia

## Abstract

**Introduction:**

Organic solvent-induced psychosyndromes (OSP) constitute a major occupational health concern, particularly in industrial sectors involving frequent use of chemical products. This neurotoxic disorder can have significant consequences on workers’ health and their ability to maintain professional activity.

**Objectives:**

This study aims to analyze the epidemiological, clinical, and occupational characteristics of organic solvent-induced psychosyndromes diagnosed in the workplace, as well as to evaluate their impact on work fitness.

**Methods:**

A retrospective study was conducted based on medical records collected at the Institute of Occupational Health and Safety (ISST) in Tunisia during a period of 5 years from 2019 until 2024. Socio-professional data, risk factors, diagnoses, progression, and fitness decisions were analyzed with a focus on OSP cases. The severity of the psychosyndrome was assessed using the Raleigh classification and the WHO criteria.

**Results:**

The study identified 11 cases of organic solvent-induced psychosyndromes. The average age of patients was 41 years. The gender distribution showed 6 women and 5 men.

The professional sectors were distributed as follows: chemical industry (n=4), textile industry (n=3), electronics (n=2), and other sectors (n=2).

The main solvents involved were xylene (n=7), toluene (n=6), hexane (n=5), and cyclohexane (n=4), with multiple exposures in 8 cases. The average duration of occupational exposure before diagnosis was 14 years ranging from 5 to 30 years. An ineffectiveness of protective measures was found in 6 patients.

The most frequently reported symptoms included memory disorders (n=9), attention disorders (n=8), and mood disorders (n=7).

The most common type of psychosyndrome was type 2B (n=8).

Regarding work fitness, nine cases required removal from solvent exposure, including six through job transfer and three through workplace accommodation. Two cases were declared unfit for their current position.

**Conclusions:**

This study highlights the impact of organic solvent-induced psychosyndromes on workers’ health and emphasizes the need for better protective measures, early screening, and regular medical monitoring.

**Disclosure of Interest:**

None Declared

